# Information-Driven Integrated Healthcare: An Analysis of the Cooperation Strategy of County Medical Community Based on Multi-Subject Simulation

**DOI:** 10.3390/healthcare11142019

**Published:** 2023-07-13

**Authors:** Changqi Dong, Jida Liu, Jianing Mi

**Affiliations:** School of Management, Harbin Institute of Technology, Harbin 150001, China; 21b910041@stu.hit.edu.cn (C.D.); mijianing@hit.edu.cn (J.M.)

**Keywords:** county medical community, integrated healthcare, digital governance, informatization construction, multi-subject simulation

## Abstract

The fragmentation and uneven quality of primary medical resources in China call for a deepening of integrated healthcare reform. China is promoting its county medical community (CMC) reform on a large scale in county-level administrative regions to promote the integration of local primary healthcare systems through information technology, which is consistent with the current trend of the digital governance era. Considering that the construction of a county medical community involves collaborative relationships between multi-level subjects, the evolutionary game theory was adopted in this study to construct a game model between the lead hospital of a CMC and primary healthcare institutions, and then the incentives of government department support were introduced to analyze the behavioral evolution of these three subjects. Taking into account the uncertainty of the real-life environment and information transformation, white Gaussian noise was introduced as a random disturbance term, and a numerical simulation was performed. In the two-subject model we focus on four parameters: information and management authority ceded by the primary healthcare institutions, integration coefficient of CMC information construction, intensity factor of information integration in the CMC, and medical resources delivered by the lead hospital. In the three-subject model we focus on three parameters: information and portion of authority ceded by the primary healthcare institutions and government departments, policy effect coefficient of CMC construction, and intensity of government departments’ support for CMC construction. The simulation results show that there is a positive incentive for the concession of management power and information from the primary healthcare institutions to the lead hospital, but further determination of empowerment boundaries is needed. The lead hospital can improve the balance of medical resources in the county through the downward transfer of medical resources, but long-term resource delivery may inhibit the enthusiasm of the lead hospital. An improvement in the information integration intensity of the CMC can promote the efficient flow of information and knowledge and enhance the organizational closeness of the county medical community. At the same time, the integration of CMC information construction reduces the cost of collaboration among medical community members and streamlines and consolidates business modules, which can promote more efficient use of medical resources. The government departments’ policies and funds provide obvious incentives to the lead hospital and primary healthcare institutions, but there is a need to explore appropriate financial payment ratios to balance the government’s financial pressure.

## 1. Introduction

Current global healthcare services are characterized by a comprehensive integration of digital transformation [1,2]. Particularly in the wake of the COVID-19 crisis, there has been a renewed focus on the performance and value of healthcare systems. Integrated healthcare services aim to enhance the continuity of healthcare services by addressing the fragmentation of the healthcare system and strengthening the coordination among different healthcare services within it [3]. Various integrative healthcare exploration practices are being conducted worldwide, including strategies such as integrated care pathways, improved governance models, integrated interventions, collaborative care, and integrated health services [4]. The reform of integrated healthcare occurs not only within health institutions but also in close connection with other public sectors, making it necessary to pay attention to cross-departmental partnerships in the promotion of integrated healthcare reform and, from the perspective of complex public affairs, governance can be called “wicked problem” [5,6,7]. In the context of building a comprehensive healthcare delivery reform, China has attempted to build a medical service alliance called the county medical community (CMC). The county medical community is a kind of institutional innovation of the Chinese government to allow the integration of medical resources and services, like a close medical organization alliance.

The county medical community model is derived from the practice of integrated healthcare, which is an alliance or a group of several healthcare institutions coming together to provide a full range of healthcare services to patients through complementary advantages and rational allocation of resources among healthcare providers, while simultaneously improving the quality and reducing the cost of services to promote efficient use of healthcare resources. The primary healthcare system in China has been significantly improved since the healthcare reform; however, there are still challenges, such as the uneven distribution of healthcare resources, the significant differentiation in the quality of healthcare services provided, and the weak financing capacity of healthcare institutions [8,9], which are the reasons for exploring the construction of the CMC model. In China’s medical institution system, public hospitals are divided into different tiers according to the administrative region they belong to. There are differences in operational efficiency among different levels of public hospitals, and studies have shown that this stems from a variety of factors, including service capacity, organizational structure, infrastructure, and administrative affiliation [10,11,12], especially among county hospitals, township health centers, and village health clinics. The Chinese government has advocated for the formation of medical communities in counties in the context of emphasizing the provision of integrated healthcare services to change the uneven allocation of healthcare resources and the weak capacity of primary healthcare services [13]. The Chinese government proposed the formation of medical communities in county-level administrative regions with a focus on exploring an integrated county–rural medical management system in which county hospitals act as the leaders, township health centers act as the hubs, and village health offices act as the foundation, thereby forming a mechanism for the division of labor and collaboration among county-level and rural medical institutions and building a three-tier medical service system [14].

The State Council of China promulgated the policy document “Guidance on Promoting the Construction and Development of Medical Consortium” in April 2017, which kicked off the CMC reform, and Zhejiang, Shanxi, and Anhui provinces carried out early pilot work in the construction of county-level medical communities. The construction of pilot county-level medical communities in these regions was mainly carried out in order to break the existing division between county-level and rural medical and health institutions in counties, to form a medical community model with a unified legal entity, implement unified management among closely linked medical and health institutions at all levels in these counties, and coordinate the distribution of interests among them; the aim was to accelerate the improvement in rural primary medical and health service capacity and promote the development of graded diagnosis and treatment [14,15,16]. The construction of the CMC reform in these Chinese provinces has gradually led to the development of a model of medical communities with knowledge characteristics, such as achieving the integration of information systems from all member units of a medical community [17]. The widespread use of information technology can facilitate efficient collaboration among healthcare institutions and rapid transfer of medical information and knowledge [18]. The capability of digitization and information technology in healthcare delivery to achieve resource integration and promote efficient collaboration and use of information has been gradually recognized [19]. It can be said that information technology offers the possibility of a ubiquitous healthcare service, which offers a feasible way for primary healthcare institutions to improve their healthcare service capacity [20]. However, primary healthcare organizations, which are responsible for a large number of complicated daily healthcare services, are still lacking in terms of the use of health information technology [21]. The construction of the CMC reform is aimed at filling this gap, and most of our known cases of CMC have been built through the use of information technology to allow different healthcare institutions to form close new alliances [13]. The information transformation of the CMC reform requires the formation of management mechanisms, such as unified medical and health resource deployment, unified accounting of medical service costs, unified performance assessments of member units, and unified medical insurance payments and salary distribution among the alliance organizations, which involves coordination and harmonization among different institutional entities [22].

The existing literature on medical communities and the integration of health services focuses on the integration efficiency assessment and integration models used in different medical fields. In terms of the efficiency assessment, Yin et al. calculated and compared the service efficiency of public hospitals under different administrative divisions based on the DEA model [12]. Sun et al. used questionnaires and cluster analysis to analyze the progress of the county medical community reform in 2019 from three aspects: resource integration, management synergy, and incentive constraints [23]. Sheiman and Shevski assessed the integration of healthcare services in Russia in terms of team coordination and continuity of services and concluded that strengthening financial incentives is an effective means of promoting integration [24]. Chen et al. empirically tested whether IT infrastructure significantly affects the service performance and financial performance of telehealth services using a model with mediating effects [25]. However, some scholars have shown that the modernization of healthcare services in a complex and rapidly changing environment is not straightforward and requires constant attention on the different strategies of action adopted by different subjects [26]. In terms of integration models used in different medical fields, a lack of coordinated leadership, a lack of harmonized funding sources, and a lack of training of health workers have caused sexual and reproductive health integration to be ineffective in health systems in sub-Saharan Africa [27]. Some scholars have also focused on how to effectively integrate health services with social services or mental health services [28]. The integration of healthcare services in the Eastern Mediterranean region, on the other hand, demonstrates the importance of governmental support and coordination of health resources among different institutions for integrated care [29]. From the existing studies, it can be seen that a discussion gap exists in the construction of county-level medical communities from the perspective of interorganizational relationships, especially regarding the choice of strategies at different levels of hospitals in the context of information technology use. Furthermore, the role of the government sector in promoting integrated healthcare needs to be explored. An evolutionary game approach is highly appropriate for systematically exploring interorganizational interactions and has been applied to different topics in the healthcare field, such as healthcare PPP projects [30], public health emergencies [31], cooperation between urban and rural medical institutions [32], online health communities [33], hierarchical diagnosis and treatment systems [34], and so on. The system dynamics approach also works well in solving complex dynamic problems [35,36] and can be used in conjunction with evolutionary game methods. Scirè system dynamics modeling assessed the relationship between social interactions, and the behavior of public institutions in the context of an infectious disease outbreak in Italy [37]. However, the traditional evolutionary game theory is rather deterministic in its assumption about the game environment. The complexity and uncertainty of information construction and digital transformation have been revealed in previous studies [38,39], and the interference of complex stochastic environments in this process needs to be further taken into account.

**Figure 1:**
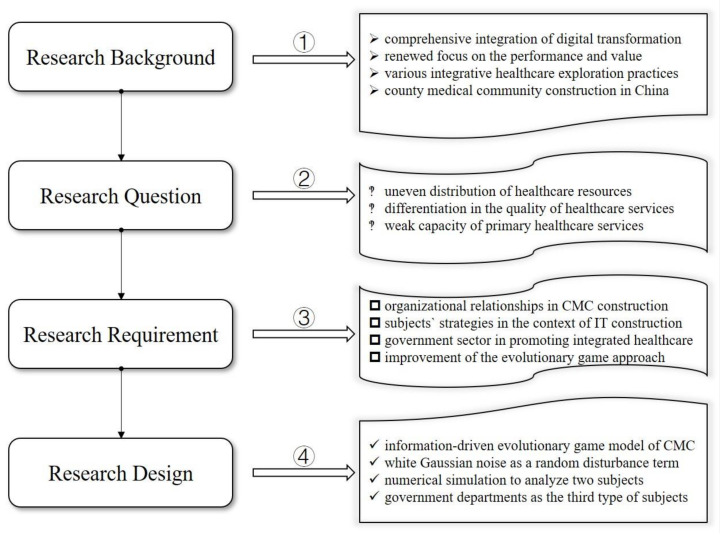
Research design flowchart.

The flowchart of our research design is shown in Figure 1. To further analyze the dynamic interorganizational interactions of the CMC reform in the context of information construction, and to explore the change paths of different subject strategies under the influence of many factors, this paper attempts to construct an information-driven stochastic evolutionary game model for the CMC reform. Firstly, a numerical simulation was used to analyze the game strategy changes of two subjects, the lead hospital of the CMC and primary healthcare institutions, and then government departments were introduced as the third type of subjects, resulting in the construction of a tripartite stochastic evolutionary game model. Overall, we used game theory and numerical simulation to explore how to improve and enhance the construction of the CMC.

## 2. Research Problem Description and Modeling Design

### 2.1. Research Problem Description

In China, healthcare services are mainly provided by government-established public hospitals. The healthcare delivery system is matched to the administrative hierarchy of a region. In other words, the administrative hierarchy determines the quantity and quality of the healthcare supply a city receives, which leads to people wanting to go to big cities for medical treatment. In the county-level administrative regions, the supply side of medical and health services mainly includes county-level public hospitals, township health centers, and village health clinics. County-level public hospitals integrate the best medical resources in a region. Township health centers and village health clinics are collectively referred to as primary healthcare institutions and are responsible for the daily medical and healthcare services for residents of villages and towns. However, differences in economic development and public services among counties lead to the possibility that residents may travel to other counties or to higher administrative levels for medical care, which means that local healthcare services are not utilized or even recognized by local residents. It is in this context that the county medical community model is born. The county medical community model is an integrated healthcare regional alliance guided by the government, led by county-level public hospitals, and involving primary healthcare institutions. The lead hospital of a CMC is able to improve the business processes of a county’s healthcare services by integrating different levels of medical services through information technology.

There are two main entities involved in this process: the lead hospital of the CMC and primary healthcare institutions. The lead hospital of the CMC is the main body responsible for basic public health services in the county. It can interface with government departments and unify the management of public health resources within the medical community. The lead hospital also supervises member units as they complete the tasks of basic public health services, and it conducts performance evaluations and benefit distributions to member units. It focuses on the treatment of acute and critically ill patients and upward referral services for difficult and complex diseases and coordinates the management of disease prevention and control within the medical community, with a focus on providing successive medical and health services for patients with chronic diseases with clear diagnoses and stable conditions and patients in recovery. The information construction of the CMC is the of use of information technology to extend organizational boundaries to achieve medical resource integration and medical business synergy. The lead hospital is responsible for promoting the use of information technology within the CMC, while the primary healthcare institutions cede their medical service information and management authority to the lead hospital. The lead hospital and primary healthcare institutions have a complex cost–benefit relationship in the construction of the CMC, and to accurately portray this relationship, we made some basic assumptions before modeling. It should be added that the model we developed is not only applicable to the Chinese practice scenario, and although the two subjects of our study originate from the Chinese healthcare system, they are also applicable in other countries. An example is the Partners Health Care System (PHS) in the United States.

### 2.2. Research Assumptions and Parameter Settings

**Assumption** **1.** *In the construction of the CMC, both the lead hospital and primary healthcare institutions have bounded rationality and are in an environment of information asymmetry. The game strategies between the lead hospital and the primary healthcare institutions in the CMC continue to adjust as the cost–benefit comparison changes over time. The lead hospital can either choose to build the county medical community’s organization efficiently and actively promote information technology reform to promote the county’s medical integration, or it can choose to build the medical community inefficiently, i.e., the set of game strategies for the lead hospital of CMC is {Efficient construction, Inefficient construction}. Let the probability that the lead hospital chooses to build the CMC efficiently be x and the probability that it builds the CMC inefficiently be 1 − x, 0 ≤ x ≤ 1. Since participation in the CMC is voluntary, primary healthcare institutions can choose either active or passive participation, i.e., the set of game strategies for primary healthcare institutions is {Active participation, Passive participation}. Let the probability of active participation by primary healthcare institutions be y and the probability of passive participation be 1 − y, 0 ≤ y ≤ 1.*

**Assumption** **2.** *To promote the construction of health service informatization in county medical communities, the lead hospital of a CMC needs to unify the information platforms of primary healthcare institutions and realize the integration of information adoption standards, information exchange standards, and information exchange methods of different medical community units [17]. This can facilitate the interconnection of medical and health information within the county and enable closed-loop medical and health services. In this process, the lead hospital of the CMC needs to pay not only the financial cost but also the cost of communication, the time cost of collaboration, etc. [18]. This can be collectively referred to as the information integration cost that the lead hospital needs to pay to promote the reform of the CMC, which is known as I. In addition, the lead hospital of the CMC needs to export management, technology, equipment, training, and other resources to primary healthcare institutions; provide routine business training and standardized guidance to township health centers; and carry out the exchange and delivery of medical resources in information construction [21]. It is assumed that the medical resources sent down from the lead hospital to the primary healthcare institutions are U. However, when the primary healthcare institutions do not actively participate in the construction of the CMC, the medical resources used and delivered by the lead hospital are not effectively absorbed. Thus, it is assumed that the absorption ratio of the primary healthcare institutions to the delivered healthcare resources is ε, which is effective when the primary healthcare institutions passively participate in the construction of the CMC.*

**Assumption** **3.** *Primary healthcare institutions joining a CMC need to cede their internal authority over the information and management of healthcare services because a CMC is a tight-knit governance structure [16]. It is assumed that the information and management authority ceded by the primary healthcare institutions to the lead hospital of the CMC is Tp. The more integrated the information construction of the medical community is, the more information and power the lead hospital of the CMC obtains from the primary healthcare institutions. As compensation, the primary healthcare institutions actively participating in the CMC are able to receive performance allocation, R, from the lead hospital, and R is amplified by the policy effects of the medical community’s construction. Due to the limited medical services and resources offered by primary healthcare institutions, patients may choose to visit hospitals in other regions or at higher levels. A decrease in attendance can result in a loss of health insurance funds, which can affect the income of primary healthcare institutions [15]. It is assumed that there is a loss of primary healthcare institutions, D, due to decreased attendance and subsequent loss of health insurance funds. The construction of the CMC is intended to restore the county’s medical attendance attrition rate and to avoid the flow of health insurance funds to other administrative regions, thus affecting the healthcare performance of the region. Thus, when primary healthcare institutions actively participate in the construction of healthcare communities, the loss D is reduced by the policy effect of the construction of healthcare communities.*

**Assumption** **4.** *Due to the differences in financial resources and infrastructure development, hospitals at different levels will have different levels of healthcare delivery capacity [40]. Assuming that the medical service capacity of the lead hospital of the CMC is Sl and the medical service capacity of the primary healthcare institutions is Sp, they will both be enhanced by the integration of information construction in the medical community.*

**Assumption** **5.** *The improvement in the information technology level of the CMC mainly relies on the lead hospital. Through the management center of the CMC, the lead hospital vertically and uniformly manages the personnel, finance, medical business, pharmacy business, medical insurance fund, information system, Chinese medicine, disease prevention and control, maternal and child health, scientific research, and logistic service support of each institution under its jurisdiction. It is assumed that the intensity factor of the lead hospital when facilitating information integration in the CMC is ξ. The intensity factor of information integration affects both the lead hospital’s information integration cost, I, and the sunk cost of the delivery of medical resources, U, as well as the information and management power, Tp, ceded by the primary healthcare institutions.*

**Assumption** **6.** *The integration level of the CMC’s information construction reflects the degree of integration of medical resources in the county, and it will affect the healthcare service capacity of medical institutions. Due to information technology construction, the functions of different hospitals are integrated under the unified organizational structure of the medical community, and the medical service business achieves unified planning, management, and benefit distribution. In other words, the integration of the CMC’s information construction can improve the medical service capacity of each hospital [14,17]. The integration coefficient of the CMC’s information construction is assumed to be π, which significantly affects the medical service capacity of the lead hospital and primary healthcare institutions. When the lead hospital and primary healthcare institutions in the CMC choose an aggressive strategy, both their medical service capabilities, Sl and Sp, are enhanced by the integration level of the CMC’s information construction.*

**Assumption** **7.** *A county’s medical service capacity has a significant impact on patient attendance rates. The formation of a CMC can reduce medical attendance attrition rates and reduce the loss of health insurance funds, an effect known as the policy effect of CMC construction [41]. The policy effect coefficient of CMC construction is assumed to be v, which can affect the loss D and the performance allocation R. Either the lead hospital or the primary healthcare institutions choosing to actively promote information technology in the medical community can increase the healthcare performance of the county, thus generating a good policy effect. For example, the attendance rate in Tianchang County, Anhui province, has surpassed 90%, and the county attendance rates in Yanhu County, Shanxi province, and Deqing County, Zhejiang province, have increased by 17.5% and 9%, respectively* [41].

The symbolic representation and range of parameters set up for Assumptions 1 to 7 are shown in Table 1.

### 2.3. Payoff Matrix and Modeling Steps for Medical Community Construction

The payoff matrix is a tool used in game theory to show the gains and losses of individual agents under different strategy combination scenarios. Table 2 shows the payoff matrix of the lead hospital and primary healthcare institutions in the construction of a CMC based on Assumptions 1 to 7. The matrix contains the benefit formulas for these two subjects under four strategy scenarios: (*x*, *y*), (*x*, 1 − *y*), (1 − *x*, *y*), and (1 − *x*,1 − *y*).

We constructed replication dynamics equations for the lead hospital and primary healthcare institutions based on the payoff matrix, which reflects the agents adapting to and learning from the behaviors of others and changing their own strategies.

The expectation expressions for the lead hospital of the CMC choosing to build efficiently and the primary healthcare institutions choosing to participate actively are, respectively, as follows:

(1) $$E_{1}^{\mathrm{LH}}=y\left[-I-U+\left(1+\pi \right)Sl+\left(1+\pi \right)\mathrm{Tp}\right]+\left(1-y\right)\left[-I-U+\left(1+\pi \right)\mathrm{Sl}+\left(1+\pi \right)\xi \mathrm{Tp}\right]$$

(2) $$E_{1}^{\mathrm{PHI}}=x\left[-\mathrm{Tp}+\left(1+\pi \right)\mathrm{Sp}+\left(1+v\right)R+U-\left(1-v\right)D\right]+\left(1-x\right)\left[-\mathrm{Tp}+\left(1+\pi \right)\mathrm{Sp}+\left(1+v\right)R+\xi U-\left(1-v\right)D\right]$$

The expectation expressions for when the lead hospital of the CMC chooses to build inefficiently, and for when the primary healthcare institutions choose to participate passively, are, respectively, as follows:

(3) $$E_{2}^{\mathrm{LH}}=y\left[-\xi \left(I+U\right)+\mathrm{Sl}+\mathrm{Tp}\right]+\left(1-y\right)\left[-\xi \left(I+U\right)+\mathrm{Sl}+\xi \mathrm{Tp}\right]$$

(4) $$E_{2}^{\mathrm{PHI}}=x\left[-\xi \mathrm{Tp}+\mathrm{Sp}+\varepsilon U-\left(1-v\right)D\right]+\left(1-x\right)\left[-\xi \mathrm{Tp}+\mathrm{Sp}+\varepsilon \xi U-D\right]$$

The average expected benefits for the lead hospital and the primary healthcare institutions in the CMC are as follows:

(5) $$\bar{E^{\mathrm{LH}}}=xE_{1}^{\mathrm{LH}}+\left(1-x\right)E_{2}^{\mathrm{LH}}$$

(6) $$\bar{E^{\mathrm{PHI}}}=yE_{1}^{\mathrm{PHI}}+\left(1-y\right)E_{2}^{\mathrm{PHI}}$$

The replication dynamic equations for the lead hospital of the CMC and the primary healthcare institutions are calculated as follows:

(7) $$L\left(x\right)=\frac{\mathrm{dx}}{\mathrm{dt}}=x\left(U_{1}^{\mathrm{LH}}-\bar{U^{\mathrm{LH}}}\right)=x\left(1-x\right)\left(U_{1}^{\mathrm{LH}}-U_{2}^{\mathrm{LH}}\right)$$

(8) $$P\left(y\right)=\frac{\mathrm{dy}}{\mathrm{dt}}=y\left(U_{1}^{\mathrm{PHI}}-\bar{U^{\mathrm{PHI}}}\right)=y\left(1-y\right)\left(U_{1}^{\mathrm{PHI}}-U_{2}^{\mathrm{PHI}}\right)$$

We obtained the replicated dynamic system of the evolutionary game between these two subjects of the information-driven CMC construction according to Equations (7) and (8):

(9) $$\left\{\begin{array}{l} L\left(x\right)=x\left(1-x\right)\left[\left(\xi -1\right)\left(I+U\right)+y\pi \left(1-\xi \right)\mathrm{Tp}+\pi \left(\mathrm{Sl}+\xi \mathrm{Tp}\right)\right]\\ P\left(y\right)=y\left(1-y\right)\left[\pi \mathrm{Sp}+\left(1+v\right)R+\left(\xi -1\right)\mathrm{Tp}+\left(1-\varepsilon \right)\xi U+\left(1-\varepsilon \right)\left(1-\xi \right)\mathrm{Ux}+\left(1-x\right)\mathrm{vD}\right] \end{array}\right.$$

## 3. Simulation of a Two-Subject Stochastic Evolutionary Game for Information-Driven CMC Construction

### 3.1. Construction of a Stochastic Evolutionary Game System for Information-Driven CMC Construction

The process of informatization and digitization is not stable or controllable, and the uncertainty of information technology absorption and application produces random interference in the information reform of the CMC, so we considered the addition of disturbance factors in the model to simulate a random environment [22]. In this study, we constructed a stochastic evolutionary game system between the lead hospital of the CMC and the primary healthcare institutions by introducing white Gaussian noise into the game model for information-driven CMC construction and obtained a system of nonlinear *Itô* stochastic differential equations:

(10) $$\left\{\begin{array}{l} \mathrm{dx}\left(t\right)=\left[\left(\xi -1\right)\left(I+U\right)+y\pi \left(1-\xi \right)\mathrm{Tp}+\pi \left(\mathrm{Sl}+\xi \mathrm{Tp}\right)\right]x\left(t\right)\mathrm{dt}+\sigma x\left(t\right)d\omega \left(t\right)\\ \mathrm{dy}\left(t\right)=\left[\pi \mathrm{Sp}+\left(1+v\right)R+\left(\xi -1\right)\mathrm{Tp}+\left(1-\varepsilon \right)\xi U+\left(1-\varepsilon \right)\left(1-\xi \right)\mathrm{Ux}+\left(1-x\right)\mathrm{vD}\right]y\left(t\right)\mathrm{dt}+\sigma y\left(t\right)d\omega \left(t\right) \end{array}\right.$$

In this system of equations, $\omega \left(t\right)$ is a one-dimensional standard Brown motion, which is an irregular motion with stochastic phenomena and, thus, can effectively portray the interference between the lead hospital and the primary healthcare institutions via stochastic factors. When the step size h is greater than 0, the increment △ω(t)=[ω(t+h)−ω(t)] follows a normal distribution $N(0,\sqrt{h})$. $d\omega \left(t\right)$ is white Gaussian noise; $\sigma x\left(t\right)d\omega \left(t\right)$ and $\sigma y\left(t\right)d\omega \left(t\right)$ are the random disturbance terms for the lead hospital and the primary healthcare institutions of the CMC, respectively; and σ is the random disturbance intensity.

Since the equations in the system of Equation (10) are nonlinear *Itô* stochastic differential equations, they do not need to be solved analytically and can be solved via stochastic Taylor expansion [42]. When $t_{0}=0$ and $t\in \left[0,T\right]$, we can divide the interval $t\in \left[0,T\right]$ into $0=t_{0}<t_{1}<t_{2}<\ldots <t_{N}=T$, and the average step size is $t_{N}=\mathrm{nh}$, n=1,2,3,…,N. If we let $x\left(t_{0}\right)=x_{0}$ and $y\left(t_{0}\right)=y_{0}$, then $x_{0},y_{0}\in R$. The Forward Euler Method was used to expand the stochastic game system (10) and obtain the system of Equation (11), according to which the simulation analysis was performed:

(11) $$\left\{\begin{array}{l} x_{n+1}=x_{n}+x\left[\left(\xi -1\right)\left(I+U\right)+y\pi \left(1-\xi \right)\mathrm{Tp}+\pi \left(\mathrm{Sl}+\xi \mathrm{Tp}\right)\right]h+\Delta \omega_{n}\sigma x(n)\\ y_{n+1}=y_{n}+y\left[\pi \mathrm{Sp}+\left(1+v\right)R+\left(\xi -1\right)\mathrm{Tp}+\left(1-\varepsilon \right)\xi U+\left(1-\varepsilon \right)\left(1-\xi \right)\mathrm{Ux}+\left(1-x\right)\mathrm{vD}\right]h+\Delta \omega_{n}\sigma y\left(n\right) \end{array}\right.$$

### 3.2. Numerical Simulation Analysis of the Two-Subject Stochastic Evolutionary Game Model for Information-Driven CMC Construction

We wrote the code for the numerical simulation based on the system of Equation (11), which allowed us to obtain a more intuitive view of the evolutionary trajectory of the entire game system. We used Python 3.9 tool to write the simulation code and run the program. And we used Origin 2021 to output the simulation image. Since real data are difficult to obtain and process, we made random assumptions and estimates for the data used in the numerical examples. Although these data are not actual scenarios, they have some reference significance for theoretical studies, and the values of these set parameters can be used to further analyze the game process presented by the lead hospital and primary healthcare institutions under the influence of different game factors. The values of the simulation parameters were determined by referring to the related literature [32,33,34], where the healthcare problem has been studied using a similar approach. The simulation parameter values were set as follows: *I* = 26, *U* = 5, *Sl* = 14, *Tp* = 12, *R* = 3, *Sp* = 2, *D* = 25, *v* = 0.5, *π* = 0.5, *ε* = 0.5, and *ξ* = 0.5. When we analyzed the influence of key parameters on the evolutionary trend of the stochastic game system in Section 3.2.1, Section 3.2.2, Section 3.2.3 and Section 3.2.4, we focused on the information and management authority ceded by the primary healthcare institutions *Tp*, the integration coefficient of CMC information construction *π*, the intensity factor of information integration in the CMC *ξ*, and the medical resources used and delivered by the lead hospital *U*.

#### 3.2.1. The Information and Management Authority Ceded by the Primary Healthcare Institutions *Tp*

**Figure 2:**
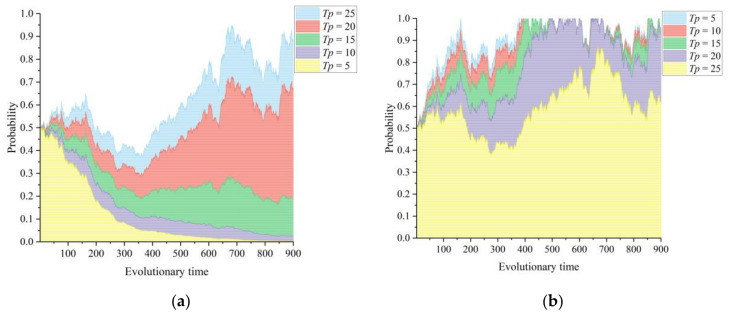
Influence of the information and management authority ceded by the primary healthcare institutions *Tp* on the evolutionary paths of the two subjects: (**a**) lead hospital of the CMC and (**b**) primary healthcare institutions.

Figure 2a,b show the dynamic effects of different levels of information and management authority ceded by the primary healthcare institutions depending on the game strategies chosen by the lead hospital and the primary healthcare institutions in the CMC, respectively. The information and management authority ceded by the primary healthcare institutions *Tp* is taken to be 5, 10, 15, 20, and 25. Medical information and management authority are the most central and exclusive resources for primary healthcare institutions. Medical community reform is actually an integration of scattered and independent medical resources to produce an effect where the whole function is greater than the sum of its parts. The information and management authority ceded by the primary healthcare institutions is an internalization of resources that are independent of each other, thus constructing a healthcare alliance. When *Tp* = 5, the lead hospital tends to choose to build inefficiently, while the primary healthcare institutions choose to actively participate in the construction of the health community. With a gradual increase in the information and management authority ceded by the primary healthcare institutions, the lead hospital can use its influence on its members to build an organizational structure with strong relationships, thus enhancing the mobilization and linkage capacity of the alliance [43]. From Figure 2b, it can be observed that an increase in *Tp* somewhat prevented the primary healthcare institutions from participating in the construction of the CMC, which reflects their hidden concern about the loss of core resources and independence. Moreover, the strategies of the primary healthcare institutions, when participating in the CMC construction, are not definite in our gaming environment but reflect a wandering and fluctuating trend, and further incentives are needed to guide strategic behaviors. When *Tp* = 25, the willingness of the primary healthcare institutions to participate in the CMC reflects a more significant decrease compared to other values of *Tp*. This suggests that there are limits and boundaries to the concession of information and management authority by primary healthcare institutions. Therefore, in practice, a lead hospital needs to negotiate with primary healthcare institutions to define the boundaries of authority and the design of the organizational structure so as not to affect the autonomy and motivation of the member institutions.

#### 3.2.2. The Integration Coefficient of CMC Information Construction *π*

**Figure 3:**
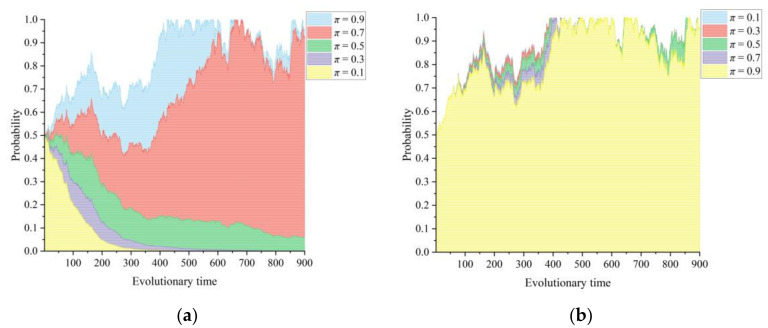
Influence of the integration coefficient of CMC information construction *π* on the evolutionary paths of the two subjects: (**a**) lead hospital of the CMC and (**b**) primary healthcare institutions.

Figure 3a,b show the dynamic effects of the integration coefficient of CMC information construction on the choice of game strategy used by the lead hospital and the primary healthcare institutions when the coefficient is taken to be 0.1, 0.3, 0.5, 0.7, and 0.9. The parameter *π* mainly reflects the degree of information sharing, structural openness, and division of labor synergy within the county healthcare community. On the one hand, it is the result of the behavior of the lead hospital and the primary healthcare institutions. On the other hand, the degree of integration of CMC information construction also affects the continuity and stability of these two subjects in the healthcare community alliance. Integrated care depends on the adequate coordination of services and collaboration among members of a healthcare community. When the level of integration of information construction in a medical community is at a low level, information standards within the alliance are not uniform, and medical data resources are difficult to integrate into a unified platform portal during transmission and processing, thus increasing the difficulty and the cost of building a medical community, which can affect the motivation of the lead hospital. Integrated information construction enhances the standardization of information sharing within the CMC, thus reducing the cost of interorganizational communication, increasing the scale and frequency of information exchange within the organization, and improving the operational efficiency of medical services. In addition, integrated information construction implies the merging and reorganization of service functions of the same type, especially the grouping of homogeneous medical services at the same level in primary healthcare institutions. By relying on the modular function of the unified information platform to perform the function of coordinating resource allocation and operation supervision, it can accurately allocate the personnel, financial, and pharmaceutical resources of each township branch hospital to improve the efficiency of medical resource utilization and stimulate the service vitality of each township branch hospital. As a result, the primary healthcare institutions will actively participate in the integration of healthcare community information construction, and the lead hospital will tend to build the CMC more efficiently.

#### 3.2.3. The Intensity Factor of Information Integration in the CMC *ξ*

**Figure 4:**
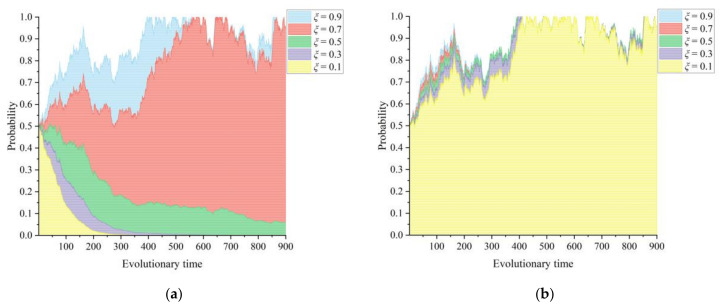
Influence of the intensity factor of information integration in the CMC *ξ* on the evolutionary paths of the two subjects: (**a**) lead hospital of the CMC and (**b**) primary healthcare institutions.

Figure 4a,b show the dynamic effects of the intensity factor of information integration in the CMC based on the game strategy choices of the lead hospital and the primary healthcare institutions in the CMC, respectively. The intensity of information integration is different at the level of information construction integration in the CMC. It is a process indicator that mainly demonstrates the degree of enthusiasm and diligence of the lead hospital and the primary healthcare institutions in promoting CMC information construction. The intensity with which the lead hospital promotes information integration in the CMC is directly reflected in the cost of information integration and the medical resources used by the primary healthcare institutions. In contrast, the intensity of information integration of the primary healthcare institutions is mainly reflected by their information and authority ceding. When the intensity of information integration in a medical community is low, the gain in healthcare services and management power obtained by the lead hospital from efficiently building the medical community is lower than the integration cost and the sunk cost of spillover healthcare resources, so the lead hospital tends to lead and build inefficiently. As the intensity of information integration increases, the stabilization strategy of the information-driven CMC construction game system gradually evolves from {Inefficient construction, active participation} to {Efficient construction, active participation}. When 0.3 < *ξ* < 0.5, the lead hospital of the CMC shifts from inefficient to efficient construction, while the strategy of the primary healthcare institutions tends to enter a positive state after some fluctuations. That is, when the intensity of information integration reaches a certain threshold, the information and knowledge within the medical community become important resources that drive its operation, and the CMC gradually forms a tight organizational structure through reorganization, which is conducive to the lead hospital’s in-depth promotion of medical community reform.

#### 3.2.4. Medical Resources Delivered by the Lead Hospital *U*

**Figure 5:**
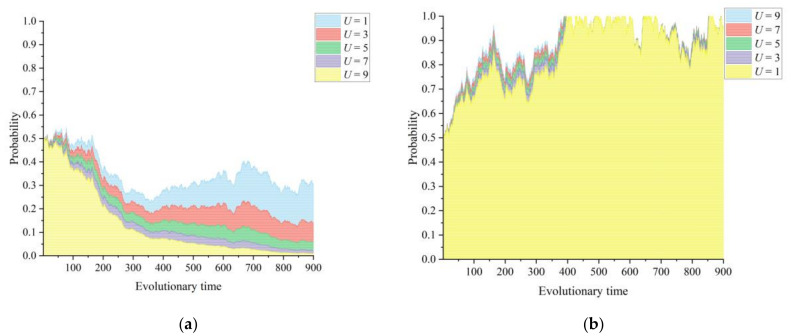
Influence of the medical resources delivered by the lead hospital *U* on the evolutionary paths of the two subjects: (**a**) lead hospital of the CMC and (**b**) primary healthcare institutions.

Figure 5a,b show the dynamic effects of the medical resources delivered by the lead hospital to the primary healthcare institutions depending on the game strategies chosen by the lead hospital and the primary healthcare institutions in the CMC. An important means of CMC construction to improve the service capacity of primary healthcare institutions is by sinking high-quality medical resources, i.e., the lead hospital assigns its own medical staff to CMC members for guidance and assistance. In particular, the core hospitals in the region share relevant numbers of experts for use by hospitals joining the CMC through internal sinking, making it easier for doctors to make direct appointments during registration when making referrals. However, the sinking of medical resources from the lead hospital to the primary healthcare institutions can, to a certain extent, dilute its own stock of medical resources and even affect its medical and health services. At the same time, the primary healthcare institutions are driven by the use of medical resources delivered by the lead hospital to improve the capacity of their healthcare services and actively participate in the construction of the medical community. Feedback from established medical community construction practices also suggests that local county hospitals appear to be overwhelmed by the long-term downward delivery of medical resources [41], and further incentives need to be provided to lead hospitals in medical communities.

## 4. Construction and Simulation of a Tripartite Stochastic Evolutionary Game Model Considering Government Incentives

The CMC reform is actually inseparable from the government’s promotion and support. In China, hospitals are institutions operating within a hierarchy in terms of medical service level; however, there is no administrative order in the relationship between upper- and lower-level hospitals, which are characterized by a business collaborative relationship. In terms of administrative relationships, public hospitals are subordinate to the health authorities at all levels of the government. However, in a medical community, only the formation of a leadership relationship between the lead hospital and the primary healthcare institutions at the upper and lower levels can enhance the closeness of the CMC and promote the integration of information construction within it. County-level health departments act as competent departments of basic public health services in a county that are in charge of improving the allocation of public health resources, strengthening the management of basic public health service projects, and promoting the organization of performance evaluations. They act in conjunction with local financial departments to implement basic public health service subsidy funds. From the construction of an established CMC, government departments and the county’s medical community form a mechanism of trust and empowerment. The medical community is given the right to dispose of funds retained from the medical insurance balance, and the lead hospital coordinates their use and is responsible for the assessment of and provision of incentives to primary healthcare institutions. Therefore, we included government departments as the third subject in the model. Personnel, organizational, and financial management rights, that formerly belonged to government departments, are now ceded to the lead hospital, while government departments also support the CMC construction.

We redesigned the basic assumptions presented in Section 2.2, the model construction in Section 2.3, and the stochastic evolutionary game simulation in Section 3 and constructed a tripartite stochastic evolutionary game model involving the lead hospital, primary healthcare institutions, and government departments, as shown in Section 4.1, Section 4.2 and Section 4.3, to conduct numerical simulations.

### 4.1. Additional Assumptions and Parameters for Considering the Government as a Subject

Continuing the seven research assumptions presented in Section 2.2, we considered government departments as the third subject and added Assumptions 8–10 as follows:

**Assumption** **8.** *In the construction of the CMC, government departments also have bounded rationality and cannot be fully informed of all the information and behaviors of individual members of a medical community. Government departments also have different options for supporting the construction of the medical community. The government will give adequate support to the construction of the CMC when it is believed that it will bring about an improvement in the level of medical services within the district and enhance its own performance. Support from government departments is prudent when they see little success in building a medical community. The set of game strategies for the primary healthcare institutions in the CMC is {Adequate support, Prudent support}. Let the probabilities of the government choosing adequate support and prudent support be z and 1 − z, respectively, where 0 ≤ z ≤ 1.*

**Assumption** **9.** *Traditional hospital management is within the administrative jurisdiction of government departments. Hospitals do not have the right to independently hire personnel and make salary decisions. Hospital management and medical staff are included in the unified and established management of institutions. In general, a high degree of administration affects the motivation of hospitals, and the staffing system, in particular, may affect the mobility of healthcare providers. The purpose of a county medical community is to de-administrate and break the solidified bondage of the staffing system [12]. This requires the lead hospital to have greater autonomy over the hiring of personnel and performance evaluation of primary healthcare institutions. It is assumed that the portion of authority ceded by the government departments to the lead hospital to support the construction of the CMC is Tg. The higher the integration coefficient of the CMC information construction is, the more power the lead hospital obtains as conceded by the government departments. At the same time, the intensity factor of information integration in the CMC also affects the management power ceded by the government departments.*

**Assumption** **10.** *Government departments will financially support the construction of a CMC. They allocate funds F to build the county medical community. The allocation of construction funds by the government departments is regulated by the intensity of the government departments’ support for the CMC construction, φ. When the government departments choose an adequate support strategy for the construction of the CMC, the intensity coefficient φ of the government departments’ support for the construction of the CMC takes a value of one. When the government departments choose a prudent support strategy for the CMC construction, the funds will be reduced, and the intensity coefficient φ of the government departments’ support for the construction of the CMC will be between zero and one. Of course, the construction of the CMC to promote the development of integrated medical services can improve local healthcare, thus allowing the relevant government departments to gain credibility from residents or through performance evaluations from superior governments. Therefore, let the political gain of the government departments from CMC construction be A. This will be increased by the policy effect coefficient of CMC construction and decreased by the intensity of the government departments’ support.*

The symbolic representation and range of parameters set up by the additional Assumptions 8 to 10 are shown in Table 3.

### 4.2. Revision of the Payoff Matrix and Game Model after the Introduction of Government Departments

We introduced government departments into the model and calculated the payoff matrices for the following three subjects: the lead hospital of the CMC (LH), the primary healthcare institutions (PHIs), and the government departments (GDs). Table 4 presents the benefit formulas for the three subjects under eight strategy scenarios: (*x*, *y*, *z*), (*x*, *y*, 1 − *z*), (*x*, 1 − *y*, *z*), (*x*, 1 − *y*, 1 − *z*), (1 − *x*, *y*, *z*), (1 − *x*, *y*, 1 − *z*), (1 − *x*, 1 − *y*, *z*), and (1 − *x*, 1 − *y*, 1 − *z*).

We constructed replication dynamic equations for the lead hospital, primary healthcare institutions, and government departments of the CMC based on the modified payoff matrix.

The expectation expressions for the lead hospital choosing to build efficiently, the primary healthcare institutions choosing to participate actively, and the government departments choosing to support adequately are, respectively, as follows:

(12) $$\begin{array}{l} E_{1}^{\mathrm{LH}}=\mathrm{yz}\left[-I-U+\left(1+\pi \right)\mathrm{Sl}+\left(1+\pi \right)\left(\mathrm{Tp}+\mathrm{Tg}\right)+F\right]+y\left(1-z\right)\left[-I-U+\left(1+\pi \right)\mathrm{Sl}+\left(1+\pi \right)\left(\mathrm{Tp}+\xi \mathrm{Tg}\right)+\varphi F\right]\\ +z\left(1-y\right)\left[-I-U+\left(1+\pi \right)\mathrm{Sl}+\left(1+\pi \right)\left(\xi \mathrm{Tp}+\mathrm{Tg}\right)+F\right]+\left(1-y\right)\left(1-z\right)\left[-I-U+\left(1+\pi \right)\mathrm{Sl}+\left(1+\pi \right)\xi \left(\mathrm{Tp}+\mathrm{Tg}\right)+\varphi F\right] \end{array}$$

(13) $$\begin{array}{l} E_{1}^{\mathrm{PHI}}=\mathrm{xz}\left[-\mathrm{Tp}+\left(1+\pi \right)\mathrm{Sp}+\left(1+v\right)R+U-\left(1-v\right)D\right]+x\left(1-z\right)\left[-\mathrm{Tp}+\left(1+\pi \right)\mathrm{Sp}+\left(1+v\right)R+U-\left(1-v\right)D\right]\\ +z\left(1-x\right)\left[-\mathrm{Tp}+\left(1+\pi \right)\mathrm{Sp}+\left(1+v\right)R+\xi U-\left(1-v\right)D\right]+\left(1-x\right)\left(1-z\right)\left[-\mathrm{Tp}+\left(1+\pi \right)\mathrm{Sp}+\left(1+v\right)R+\xi U-\left(1-v\right)D\right] \end{array}$$

(14) $$\begin{array}{l} E_{1}^{\mathrm{GD}}=\mathrm{xy}\left[-\mathrm{Tg}-F+\left(1+v\right)A\right]+x\left(1-y\right)\left[-\mathrm{Tg}-F+\left(1+v\right)A\right]\\ +y\left(1-x\right)\left[-\mathrm{Tg}-F+\left(1+v\right)A\right]+\left(1-x\right)\left(1-y\right)\left[-\mathrm{Tg}-F+A\right] \end{array}$$

The expectation expressions for the lead hospital choosing inefficient construction, the primary healthcare institutions choosing passive participation, and the government departments choosing prudent support are as follows:

(15) $$\begin{array}{l} E_{2}^{\mathrm{LH}}=\mathrm{yz}\left[-\xi \left(I+U\right)+\mathrm{Sl}+\mathrm{Tp}+\mathrm{Tg}+\xi F\right]+y\left(1-z\right)\left[-\xi \left(I+U\right)+\mathrm{Sl}+\mathrm{Tp}+\xi \mathrm{Tg}+\varphi \xi F\right]\\ +z\left(1-y\right)\left[-\xi \left(I+U\right)+\mathrm{Sl}+\xi \mathrm{Tp}+\mathrm{Tg}+\xi F\right]+\left(1-y\right)\left(1-z\right)\left[-\xi \left(I+U\right)+\mathrm{Sl}+\xi \left(\mathrm{Tp}+\mathrm{Tg}\right)+\varphi \xi F\right] \end{array}$$

(16) $$\begin{array}{l} E_{2}^{\mathrm{PHI}}=\mathrm{xz}\left[-\xi \mathrm{Tp}+\mathrm{Sp}+\varepsilon U-\left(1-v\right)D\right]+x\left(1-z\right)\left[-\xi \mathrm{Tp}+\mathrm{Sp}+\varepsilon U-D\right]\\ +z\left(1-x\right)\left[-\xi \mathrm{Tp}+\mathrm{Sp}+\varepsilon \xi U-\left(1-v\right)D\right]+\left(1-x\right)\left(1-z\right)\left[-\xi \mathrm{Tp}+\mathrm{Sp}+\varepsilon \xi U-D\right] \end{array}$$

(17) $$\begin{array}{l} E_{2}^{\mathrm{GD}}=\mathrm{xy}\left[-\xi \mathrm{Tg}-\varphi F+\varphi \left(1+v\right)A\right]+x\left(1-y\right)\left[-\xi \mathrm{Tg}-\varphi F+\varphi \left(1+v\right)A\right]\\ +y\left(1-x\right)\left[-\xi \mathrm{Tg}-\varphi F+\varphi \left(1+v\right)A\right]+\left(1-x\right)\left(1-y\right)\left[-\mathrm{Tg}-\varphi F\right] \end{array}$$

The average expected benefits for the lead hospital, the primary healthcare institutions, and the government departments are as follows:

(18) $$\bar{E^{\mathrm{LH}}}=xE_{1}^{\mathrm{LH}}+\left(1-x\right)E_{2}^{\mathrm{LH}}$$

(19) $$\bar{E^{\mathrm{PHI}}}=yE_{1}^{\mathrm{PHI}}+\left(1-y\right)E_{2}^{\mathrm{PHI}}$$

(20) $$\bar{E^{\mathrm{GD}}}=zE_{1}^{\mathrm{GD}}+\left(1-z\right)E_{2}^{\mathrm{GD}}$$

The replication dynamic equations for the lead hospital of the CMC, the primary healthcare institutions, and the government departments are calculated as follows:

(21) $$L\left(x\right)=\frac{\mathrm{dx}}{\mathrm{dt}}=x\left(U_{1}^{\mathrm{LH}}-\bar{U^{\mathrm{LH}}}\right)=x\left(1-x\right)\left(U_{1}^{\mathrm{LH}}-U_{2}^{\mathrm{LH}}\right)$$

(22) $$P\left(y\right)=\frac{\mathrm{dy}}{\mathrm{dt}}=y\left(U_{1}^{\mathrm{PHI}}-\bar{U^{\mathrm{PHI}}}\right)=y\left(1-y\right)\left(U_{1}^{\mathrm{PHI}}-U_{2}^{\mathrm{PHI}}\right)$$

(23) $$G\left(z\right)=\frac{\mathrm{dz}}{\mathrm{dt}}=z\left(U_{1}^{\mathrm{GD}}-\bar{U^{\mathrm{GD}}}\right)=z\left(1-z\right)\left(U_{1}^{\mathrm{GD}}-U_{2}^{\mathrm{GD}}\right)$$

We calculated and obtained the replicated dynamic system of the evolutionary game among the three subjects of the information-driven CMC construction according to Equations (21) and (23):

(24) $$\left\{\begin{array}{l} L\left(x\right)=x\left(1-x\right)\left(U_{1}^{\mathrm{LH}}-U_{2}^{\mathrm{LH}}\right)=x\left(1-x\right)\left[\left(\xi -1\right)\left(I+U\right)+y\pi \left(1-\xi \right)\mathrm{Tp}+z\pi \left(1-\xi \right)\mathrm{Tg}+\pi \left(\mathrm{Sl}+\xi \mathrm{Tp}+\xi \mathrm{Tg}\right)+\left(1-\xi \right)\varphi F\right]\\ P\left(y\right)=y\left(1-y\right)\left(U_{1}^{\mathrm{PHI}}-U_{2}^{\mathrm{PHI}}\right)=y\left(1-y\right)\left[\pi \mathrm{Sp}+\left(1+v\right)R+\left(\xi -1\right)\mathrm{Tp}+\left(1-\varepsilon \right)\xi U+\left(1-\varepsilon \right)\left(1-\xi \right)\mathrm{Ux}+\left(1-z\right)\mathrm{vD}\right]\\ G\left(z\right)=z\left(1-z\right)\left(U_{1}^{\mathrm{GD}}-U_{2}^{\mathrm{GD}}\right)=z\left(1-z\right)\left[A\left(\varphi -v+\varphi v\right)\left(\mathrm{xy}-x-y\right)+\left(\xi -1\right)\mathrm{Tg}-\left(1-\varphi \right)F+A\right] \end{array}\right.$$

Similarly, we obtained the tripartite stochastic evolutionary game system (25) by modifying the system of Equation (24) using the procedure shown in Equations (10) and (11). We carried out a numerical simulation similar to the simulation described in Section 4.3 based on the system of Equation (25):

(25) $$\left\{\begin{array}{l} x_{n+1}=x_{n}+x\left[\left(\xi -1\right)\left(I+U\right)+y\pi \left(1-\xi \right)\mathrm{Tp}+z\pi \left(1-\xi \right)\mathrm{Tg}+\pi \left(\mathrm{Sl}+\xi \mathrm{Tp}+\xi \mathrm{Tg}\right)+\left(1-\xi \right)\varphi F\right]h+\Delta \omega_{n}\sigma x(n)\\ y_{n+1}=y_{n}+y\left[\pi \mathrm{Sp}+\left(1+v\right)R+\left(\xi -1\right)\mathrm{Tp}+\left(1-\varepsilon \right)\xi U+\left(1-\varepsilon \right)\left(1-\xi \right)\mathrm{Ux}+\left(1-z\right)\mathrm{vD}\right]h+\Delta \omega_{n}\sigma y\left(n\right)\\ z_{n+1}=z_{n}+z\left[A\left(\varphi -v+\varphi v\right)\left(\mathrm{xy}-x-y\right)+\left(\xi -1\right)\mathrm{Tg}-\left(1-\varphi \right)F+A\right]h+\Delta \omega_{n}\sigma z\left(n\right) \end{array}\right.$$

### 4.3. Numerical Simulation Analysis of the Tripartite Stochastic Evolutionary Game Model for Information-Driven CMC Construction

We adjusted the simulation code according to the equation set (25) and continued the simulation value setting shown in Section 3.2 to further analyze the game process of the lead hospital, primary healthcare institutions, and government departments under the influence of different game factors. The simulation platform used the same python 3.9 as the Section 3.2, as well as using Origin 2021 to process the simulation images. The simulation parameter values were set as follows: *I* = 26, *U* = 5, *Sl* = 14, *Tp* = 12, *R* = 3, *Sp* = 2, *Tg* = 6, *F* = 6, *A* = 10, *D* = 25, *v* = 0.5, *π* = 0.5, *ε* = 0.5, *ξ* = 0.5, and *φ* = 0.5. In Section 4.3.1, Section 4.3.2 and Section 4.3.3, we present the results of the analysis of the authority ceded by the primary healthcare institutions and government departments *Tp* + *Tg*, the policy effect coefficient of CMC construction *v*, and the intensity of government departments’ support for the CMC construction *φ*.

#### 4.3.1. The Information and Portion of Authority Ceded by Primary Healthcare Institutions and Government Departments *Tp* + *Tg*

**Figure 6:**
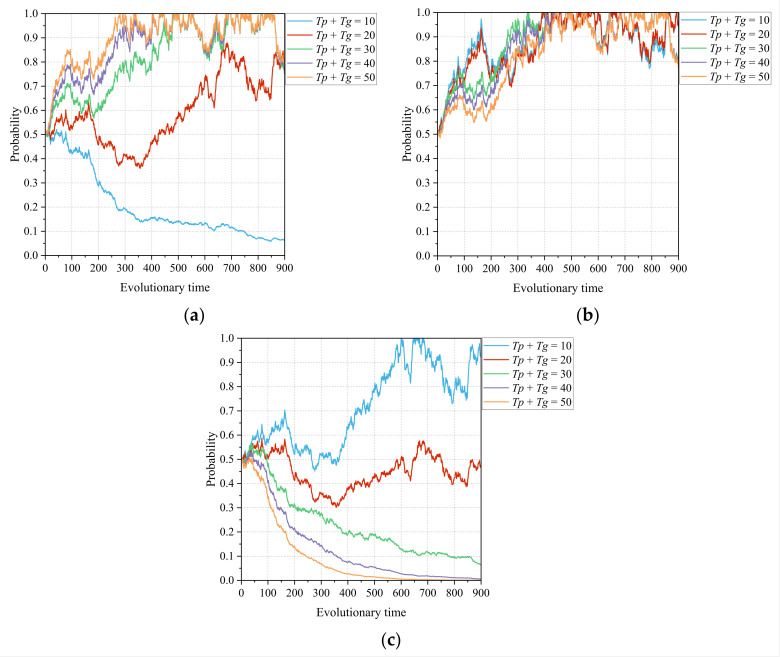
Influence of the information and portion of authority ceded by the primary healthcare institutions and government departments *Tp* + *Tg* on the evolutionary paths of the tripartite subjects: (**a**) lead hospital of the CMC, (**b**) primary healthcare institutions, and (**c**) government departments.

Figure 6a–c shows the dynamic effects of the information and portion of authority ceded by the primary healthcare institutions and government departments, depending on the choice of game strategies of the lead hospital, the primary healthcare institutions, and the government departments in the CMC. Overall, the steady-state points of the tripartite game system show a transition trajectory from (0, 1, 1) to (1, 1, 1) to (1, 1, 0), which indicates the existence of *Tp* + *Tg* values that make all three subjects choose active strategies. Concentrating excess management authority in the lead hospital would increase the centralization and autonomy of the medical alliance, which would discourage government departments from continuing to support the construction of the CMC. This means that no matter how much the government departments empower the lead hospital, the bottom line is that it must not threaten their authoritative position. In comparison with Figure 2, it can be seen that the motivation of the lead hospital and primary healthcare institutions to choose an aggressive strategy, and the efficiency with which this occurs, are substantially increased under the influence of the introducing local government incentives. In Figure 2a, the lead hospital tends to build inefficiently at *Tp* = 20 whereas, in Figure 6a, the lead hospital tends to build inefficiently when *Tp* + *Tg* = 20 and chooses to build efficiently at a faster rate when *Tp* + *Tg* takes a larger value. This suggests that the introduction of local government incentives can effectively enhance the motivation and ability of the lead hospital to promote CMC construction, a phenomenon that also applies to primary healthcare institutions. Therefore, there is a need to further explore the empowerment mechanism between the lead hospital and the government departments to find a reasonable limit for empowerment and jurisdictional boundaries.

#### 4.3.2. The Policy Effect Coefficient of CMC Construction *v*

**Figure 7:**
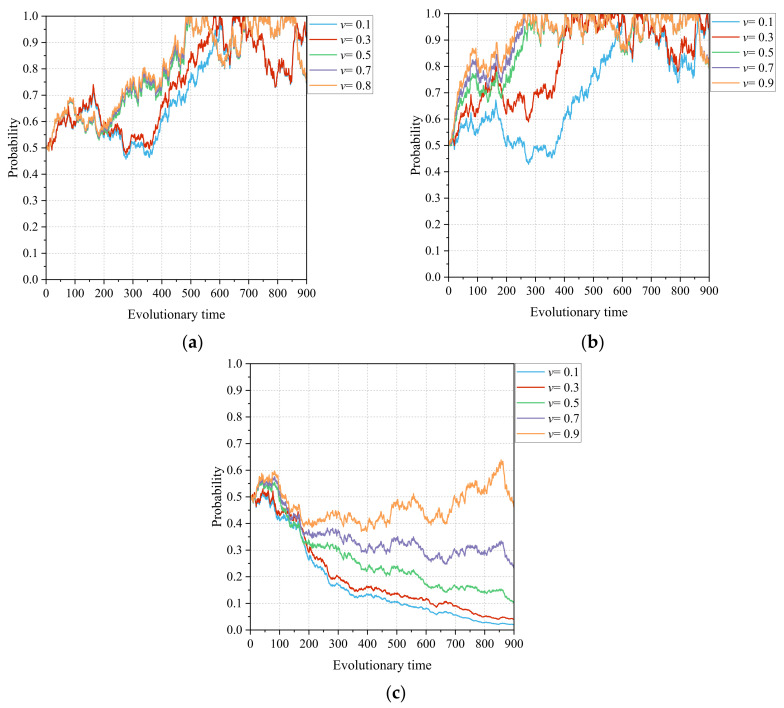
Influence of the policy effect coefficient of CMC construction *v* on the evolutionary paths of the tripartite subjects: (**a**) lead hospital of the CMC, (**b**) primary healthcare institutions, and (**c**) government departments.

Figure 7a–c show the dynamic effects of the policy effect coefficient of CMC construction depending on the game strategies chosen by the lead hospital, the primary healthcare institutions, and the government departments, respectively. Unlike other parameters, such as the integration coefficient of CMC information construction and the intensity factor of information integration in the CMC, which act on costs or losses, the policy effect coefficient directly responds to the increase and amplification of the medical and health benefits of CMC information construction for different subjects. Under the influence of the policy effect coefficient of CMC construction, the tripartite game system shows a progressive trend from (1, 1, 0) to (1, 1, 1) and, although this process is not realized, the trend is evident. In Figure 7a,b, as *v* increases from 0.3 to 0.5, the rate at which the lead hospital chooses to build efficiently also increases significantly, while the primary healthcare institutions lower the strategic process of choosing to actively participate in the construction of the medical community by nearly half. The policy incentive of CMC construction has a significant impact on the participation of different subjects in medical community construction, which constitutes a good feedback loop. On the one hand, the lead hospital and primary healthcare institutions choosing to efficiently and actively build the CMC can effectively unlock the policy dividends of integrated healthcare alliance, thus enhancing regional healthcare performance. On the other hand, the policy effects of the CMC will provide positive feedback and incentives for the participants to strengthen their behavior and, thus, form their participation practices, further deepening the closeness and synergy of healthcare community cooperation.

#### 4.3.3. The Intensity of Government Departments’ Support for CMC Construction *φ*

**Figure 8:**
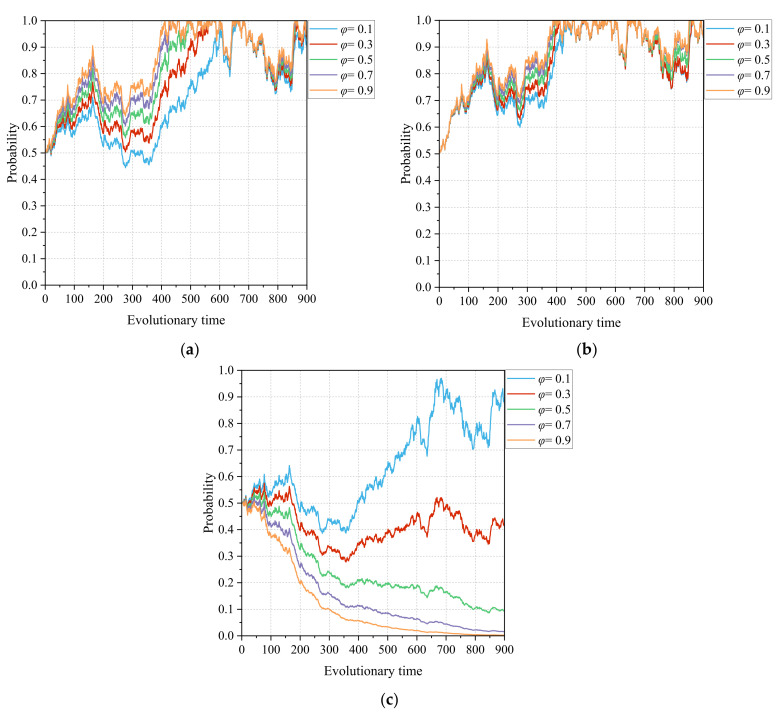
Influence of the intensity of the government departments’ support for CMC construction *φ* on the evolutionary paths of the tripartite subjects: (**a**) lead hospital of the CMC, (**b**) primary healthcare institutions, and (**c**) government departments.

Figure 8a–c show the sensitivity of the three parties in the game system, namely the lead hospital, the primary healthcare institutions, and the government departments, to the intensity of the government departments’ support for the construction of the medical community. The intensity of the government departments’ support for CMC construction *φ* is taken to be 0.1~0.9 with an interval of 0.2, and the evolution of the information-driven tripartite stochastic game system of CMC construction is shown in Figure 8. Government departments have formed a community of interest and performance with CMC in promoting information technology in the medical community. The intensity of government departments’ support affects both the policy and financial support that a CMC receives from the government and the performance of a local government in the medical community reform. For the lead hospital of a CMC, a larger *φ* indicates more incentive gains from government departments, such as special funding support for the CMC construction. The incentive of adequate construction funding can drive the lead hospital to build efficiently. At the same time, as the intensity *φ* increases, the rate at which the lead hospital chooses to build efficiently increases and fluctuates less, and the primary healthcare institutions choose to actively participate with a similar trend in strategy. Nevertheless, government departments need to allocate limited financial resources to different policy areas for guidance and incentives, which means that, although increasing the intensity of support for a CMC can yield a better health performance, government departments need to consider an appropriate ratio of support. Too much support can cause a change in the behavior of government departments, triggering a shift in their strategic choice from “adequate support” to “prudent support”.

## 5. Discussion

The hierarchy of public hospitals in China has led to the inevitable fragmentation and uneven quality of primary healthcare resources, which has made the exploration of integrated healthcare models a focus of academic research. The county medical community reform is a practical way to solve the scattering of primary medical and healthcare resources and to improve the capability of primary medical services. The promotion of information technology plays a vital role in CMC construction. County-level hospitals, township health centers, and village health clinics are integrated into a tight network of healthcare services within the cooperative framework of a county’s medical community. The realization of CMC information construction is intended to adapt to the current trend of widespread use of information technology in the era of digital governance. The application of information technology in CMC construction can improve the business processes of internal institutions, enhance business cooperation among different hospitals, and unify performance evaluation to one set of standards from the supply side of medical services. However, the process of promoting information technology in a CMC involves the coordination and adjustment of cooperative relationships among different types of organizations and hospitals at different levels. It should be added that the coordination problems involving different departments and organizations in the information construction referred to in this paper are not limited to the application scenario in China, although they originate from the practice of county medical community construction in China, and the research content of this paper is also of some significance to the construction of integrated healthcare in other countries. We will, then, organize this Section 5 in terms of both comparing related studies and developing a discussion of the main points of our work.

In this paper, we proposed 10 basic assumptions, involving the lead hospital of a CMC, primary healthcare institutions, and government departments, based on the real-life practice of information technology construction of CMCs in China and established benefit function and replication dynamic equations for each subject based on these assumptions. We introduced white Gaussian noise as a random disturbance term and constructed a stochastic evolutionary game model for two subjects, the lead hospital and the primary healthcare institutions of the medical community, and a tripartite stochastic evolutionary game model, including incentives provided by government departments, and conducted numerical simulations. This study aimed to engage in a theoretical dialogue with existing research related to healthcare collaboration on a practical basis, to compare our work with previous similar studies, and to further discuss both the research questions and the research methods. In terms of research on cooperation among healthcare institutions, traditional evolutionary game methods have been used to study areas such as hierarchical diagnosis and treatment systems [34], cooperation between urban and rural medical institutions [32], and healthcare PPP projects [30]. In the study of county healthcare communities, scholars have explored a CMC construction model [15], the impact of CMC construction on local healthcare performance [23], and factors related to the integration of the CMC [24,25,26]. Compared with these previous studies, the major differences in our work are the change in research perspective and an improvement in the research methods used. Firstly, we examined the behavioral manifestations of three heterogeneous organizations, namely the lead hospital of the CMC, the primary healthcare institutions, and the government departments, and how they influence each other during CMC information construction from the perspective of interorganizational relationships. This differs from previous research exploring how to construct a healthcare community and investigating how it turns out at the macro level. We explored how to improve factors to make different subjects work together better at the micro-subject interaction level. Secondly, although previous studies using the evolutionary game approach have also explored from the perspective of subject cooperation, the traditional evolutionary game methodology assumes a game design in a deterministic environment, and its simulation results are smooth and linear. However, such results do not quite match the complexity and uncertainty of real environments. In particular, many studies have noted that digital transformation is inherently riskier and more uncertain [38,39]. This led us to further consider the introduction of a stochastic function characterizing the disturbances of a complex environment in the traditional evolutionary game theory. Thus, our simulation results reflect obvious nonlinear and stochastic characteristics.

Specifically, the paper discusses three main aspects of the study: sectoral empowerment, resource interaction, and government incentives.

Firstly, the management of power and information concession is an important issue in the process of information technology construction in county medical communities. The concession of information and management authority by primary healthcare institutions to the lead hospital can reduce the exclusivity of decentralized healthcare resources and enable the internalization of external resources. The simulation results presented in Figure 2 show that the influence of the lead hospital on its members increases as the primary healthcare institutions cede more information and power, which allows the lead hospital to build a strong relational organizational structure in the healthcare community, thus enhancing the linkages between medical institutions. However, the concession of power can cause primary healthcare institutions to worry about losing their independent status and resource autonomy, causing them to fluctuate in and wander from active participation in the CMC construction, which highlights that designers of CMC mechanisms need to pay attention to the power boundaries and organizational structure of CMC. The simulation results presented in Figure 6 show the impact of introducing government departments’ management authority concessions on the tripartite game process. Similarly, too much concession of management authority can inhibit government departments from continuing to support the construction of the medical community. However, the willingness of primary healthcare institutions to participate is greatly enhanced by the introduction of governmental support. In general, the lead hospital needs to further explore empowerment mechanisms with primary healthcare institutions and government departments to determine an appropriate empowerment and jurisdictional boundary.

Secondly, the lead hospital of the medical community improves the overall balance of resources within the administrative region by providing high-quality medical resources to primary healthcare institutions, which is a fairly straightforward way to do so. However, this can also cause a dilution of its own stock of medical resources. In real-life practice, long-term resource exportation can result in the lead hospital gradually losing enthusiasm in participating in CMC construction, and the simulation results presented in Figure 5 also indicate this finding. On this basis, the simulation results presented in Figure 4 show that the intensity of information integration in the CMC has a more significant impact on the game outcome of information construction in the medical community. The intensity of information integration is a reflection of the level of due diligence by the lead hospital and primary healthcare institutions in promoting information transformation in the medical community. From the viewpoint of operation management, information construction can improve the lead hospital’s ability to monitor the operation of institutions within the medical community in real time. From the viewpoint of service provision, the information platform can support township health centers and county-level hospitals to provide integrated services and offer the possibility of communication and exchange of services, information, and technology among institutions [44]. The simulation results show that, with an increase in the information integration intensity in the CMC, the high level of informatization promotes the efficient flow of information and knowledge within the alliance, and the CMC gradually forms a tight organizational structure through reorganization under this strong information connection. To a certain extent, self-managed information technology construction can improve the internal operational efficiency of individual township health centers, but the improvement made to their weak business service capacity is extremely limited. Homogeneous competition still causes rural patients to flock directly to county-level hospitals instead of those in their townships. Hence, the realization of information construction integration is crucial to the advancement of a CMC, and the level of information construction integration goes hand in hand with the improvement in integrated healthcare. Integrated information construction reduces the cost of collaboration and simplifies the business modules among the members of a medical community, which can improve the efficiency of medical resource utilization.

Finally, the introduction of government departments as a third subject was further considered in this study to examine the evolution of the CMC game system. The intensity of government departmental support mainly affects the policy and financial support received for county medical community construction, which further affects the performance gains obtained by the local government in the medical community reform. In other words, government departments form a community of interest during the reform of the medical community with the lead hospital and primary healthcare institutions. The simulation results presented in Figure 8 show that the intensity of government departments’ support can drive the lead hospital to actively engage in medical community building. Moreover, as the intensity of governmental support for the CMC construction increases, the rate at which the lead hospital and primary healthcare institutions choose aggressive strategies increases, and fluctuations in their choices decrease. That is, the incentive of financial funding plays a risk-resistant and protective role for hospitals, thus promoting information technology reform [45]. Nevertheless, there is a budgetary constraint on financial resources, and government departments need to explore an appropriate allocation ratio to balance financial support in different areas. Indeed, the support of government departments is also aimed at realizing the policy effects of county medical community reform. The simulation process presented in Figure 7 shows that there is a critical and significant change in the motivation of the lead hospital and primary healthcare institutions with the policy effect, and the motivation of multiple subjects, in turn, promotes the construction of the healthcare community, forming a positive feedback loop. Therefore, it is necessary for government policies to play a guiding role in the promotion of CMC construction. The use of policy incentives can promote a plurality of subjects to form a consensus and take consistent action, which can jointly promote the reform of county medical communities.

## 6. Conclusions

The goal of building a medical community fits in with the relationship between the whole and the parts in systems thinking in philosophy. The aim of a CMC is to integrate decentralized medical resources in an administrative region, and the resulting alliance of medical service supply seeks to provide better medical and health services. This study focused on county medical community reform as a practical exploration model of integrated healthcare and used the stochastic evolutionary game approach to explore interorganizational cooperation in CMC construction, constructing a two-subject stochastic evolutionary game model for the lead hospital and primary healthcare institutions of a CMC and a tripartite evolutionary game model with the introduction of government departments. It also analyzed the effects of different factors on the stochastic evolutionary game for information-driven CMC construction through numerical simulation. The findings suggest that the cession of management power and information from the primary healthcare institutions can motivate the lead hospital to actively promote the use of information technology in the CMC, but further discussions with the primary healthcare institutions and government departments regarding the boundaries of empowerment are needed. The lead hospital of the CMC can improve the balance of medical resources in the county by providing healthcare resources to primary healthcare institutions, but long-term resource delivery will inhibit the motivation of the lead hospital. An improvement in the information integration intensity of the CMC can promote the efficient flow of information and knowledge within the county medical community and enhance organizational closeness. The integration of information construction in the CMC reduces the cost of collaboration among members and streamlines business modules, which can promote more efficient utilization of medical resources. Under the policy and financial underwriting incentives from government departments, the lead hospital and primary healthcare institutions can actively pursue CMC construction, but appropriate financial payment ratios need to be explored to balance the government’s financial pressure with the policy effect dividends of CMC construction.

## Figures and Tables

**Table 1:** Parameters, symbols, and ranges.

Symbols	Parameters	Ranges
*I*	information integration cost of the lead hospital	*I* > 0
*U*	cost of medical resources delivered by the lead hospital	*U* > 0
*Sl*	medical service capacity of the lead hospital	*Sl* > 0
*Tp*	information and management authority ceded by the primary healthcare institutions	*Tp* > 0
*R*	performance allocation of the primary healthcare institutions	*R* > 0
*Sp*	medical service capacity of the primary healthcare institutions	*Sp* > 0
*D*	loss of primary healthcare institutions due to decreased attendance	*n* > 0
*v*	policy effect coefficient of CMC construction	0 ≤ *v* ≤ 1
*π*	integration coefficient of CMC information construction	0 ≤ *π* ≤ 1
*ε*	absorption ratio of primary healthcare institutions to delivered healthcare resources	0 ≤ *ε* ≤ 1
*ξ*	intensity factor of information integration in the CMC	0 ≤ *ξ* ≤ 1

**Table 2:** The payoff matrix of the lead hospital of the CMC and primary healthcare institutions.

Game Agents and Behavioral Strategies		Lead Hospital of the CMC (LH)	
		Efficient Construction (*x*)	Inefficient Construction (1 − *x*)
Primary healthcare institutions (PHIs)	Active participation (*y*)	$-I-U+\left(1+\pi \right)\mathrm{Sl}+\left(1+\pi \right)\mathrm{Tp}$, $-\mathrm{Tp}+\left(1+\pi \right)\mathrm{Sp}+\left(1+v\right)R+U-\left(1-v\right)D$	$-\xi \left(I+U\right)+\mathrm{Sl}+\mathrm{Tp}$, $-\mathrm{Tp}+\left(1+\pi \right)\mathrm{Sp}+\left(1+v\right)R+\xi U-\left(1-v\right)D$
	Passive participation (1 − *y*)	$-I-U+\left(1+\pi \right)\mathrm{Sl}+\left(1+\pi \right)\xi \mathrm{Tp}$, $-\xi \mathrm{Tp}+\mathrm{Sp}+\varepsilon U-\left(1-v\right)D$	$-\xi \left(I+U\right)+\mathrm{Sl}+\xi \mathrm{Tp}$, −ξTp+Sp+εξU−D

**Table 3:** Additional parameters, symbols, and ranges.

Symbols	Parameters	Ranges
*Tg*	portion of authority ceded by government departments	*Tg* > 0
*F*	funds allocated by government departments for the construction of a county medical community	*F* > 0
*A*	political gains from the CMC construction	*A* > 0
*φ*	intensity of government departments’ support for the CMC construction	0 ≤ *φ* ≤ 1

**Table 4:** The payoff matrix of the lead hospital of the CMC, primary healthcare institutions, and government departments.

Game Agents and Behavioral Strategies				LH	
				Efficient Construction (*x*)	Inefficient Construction (1 − *x*)
PHI	Active participation (*y*)	GD	Adequate support (*z*)	$-I-U+\left(1+\pi \right)\mathrm{Sl}+\left(1+\pi \right)\left(\mathrm{Tp}+\mathrm{Tg}\right)+F$, $-\mathrm{Tp}+\left(1+\pi \right)\mathrm{Sp}+\left(1+v\right)R+U-\left(1-v\right)D$, $-\mathrm{Tg}-F+\left(1+v\right)A$	$-\xi \left(I+U\right)+\mathrm{Sl}+\mathrm{Tp}+\mathrm{Tg}+\xi F$, $-\mathrm{Tp}+\left(1+\pi \right)\mathrm{Sp}+\left(1+v\right)R+\xi U-\left(1-v\right)D$, $-\mathrm{Tg}-F+\left(1+v\right)A$
			Prudent support (1 − *z*)	$-I-U+\left(1+\pi \right)\mathrm{Sl}+\left(1+\pi \right)\left(\mathrm{Tp}+\xi \mathrm{Tg}\right)+\varphi F$, $-\mathrm{Tp}+\left(1+\pi \right)\mathrm{Sp}+\left(1+v\right)R+U-\left(1-v\right)D$, $-\xi \mathrm{Tg}-\varphi F+\varphi \left(1+v\right)A$	$-\xi \left(I+U\right)+\mathrm{Sl}+\mathrm{Tp}+\xi \mathrm{Tg}+\varphi \xi F$, $-\mathrm{Tp}+\left(1+\pi \right)\mathrm{Sp}+\left(1+v\right)R+\xi U-\left(1-v\right)D$, $-\xi \mathrm{Tg}-\varphi F+\varphi \left(1+v\right)A$
	Passive participation (1 − *y*)		Adequate support (*z*)	$-I-U+\left(1+\pi \right)\mathrm{Sl}+\left(1+\pi \right)\left(\xi \mathrm{Tp}+\mathrm{Tg}\right)+F$, $-\xi \mathrm{Tp}+\mathrm{Sp}+\varepsilon U-\left(1-v\right)D$, $-\mathrm{Tg}-F+\left(1+v\right)A$	$-\xi \left(I+U\right)+\mathrm{Sl}+\xi \mathrm{Tp}+\mathrm{Tg}+\xi F$, −ξTp+Sp+εξU−D, −Tg−F+A
			Prudent support (1 − *z*)	$-I-U+\left(1+\pi \right)\mathrm{Sl}+\left(1+\pi \right)\xi \left(\mathrm{Tp}+\mathrm{Tg}\right)+\varphi F$, $-\xi \mathrm{Tp}+\mathrm{Sp}+\varepsilon U-\left(1-v\right)D$, $-\xi \mathrm{Tg}-\varphi F+\varphi \left(1+v\right)A$	$-\xi \left(I+U\right)+\mathrm{Sl}+\xi \left(\mathrm{Tp}+\mathrm{Tg}\right)+\varphi \xi F$, −ξTp+Sp+εξU−D, −ξTg−φF

## Data Availability

Data sharing is not applicable.
